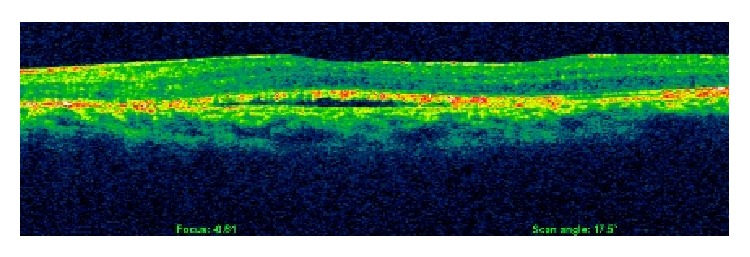# Serous Macular Detachment Secondary to Optic Pit: Surgical Treatment and Long Time Results

**DOI:** 10.1155/2016/4567840

**Published:** 2016-01-04

**Authors:** Selim Cevher, Nedime Sahinoglu-Keskek, Fikret Unal, Selahaddin Demirduzen, Huseyin Oksuz

**Affiliations:** ^1^Department of Ophthalmology, Eregli State Hospital, 42040 Konya, Turkey; ^2^Department of Ophthalmology, Adana Numune Training and Research Hospital, 01240 Adana, Turkey; ^3^Department of Ophthalmology, Hakkari State Hospital, 30010 Hakkari, Turkey

## Abstract

32-year-old Turkish male patient presented with an optic disk pit and serous macular detachment in the left eye. Spectral domain optical coherence tomography revealed serous macular detachment and retinoschisis. After vitrectomy the retina gradually flattened and vision was gradually improved. We aimed to report a case of serous macula detachment secondary to optic pit and long term result of surgical treatment.

## 1. Introduction

Optic disc pits are congenital disc abnormality secondary to a colobomatous malformation of the optic nerve head. Optic disc pit was first described by Wiethe in 1882 [1]. An optic disc pit usually appears as a solitary, oval, gray-white depression located in the inferotemporal segment of the optic disc. Acquired visual acuity loss generally is the result of the development of serous detachment of the macula, with a concomitant ophthalmoscopic appearance of the inner retina mimicking retinoschisis [2]. Among approximately 44%–66% of eyes with optic disc pits, the associated serous retinal detachment can be seen, most commonly in the second or third decade [3].

We present a case of optic disc pit associated with serous macular detachment which was successfully managed with vitrectomy. Such cases have been described in literature but long term results are rarely reported.

## 2. Case Report

**Figure 1:**
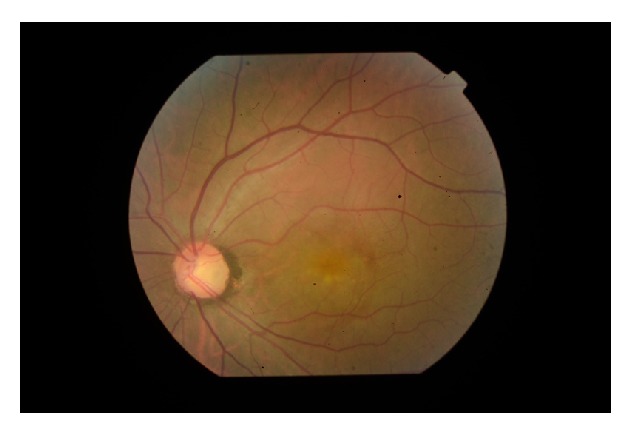


**Figure 2:**
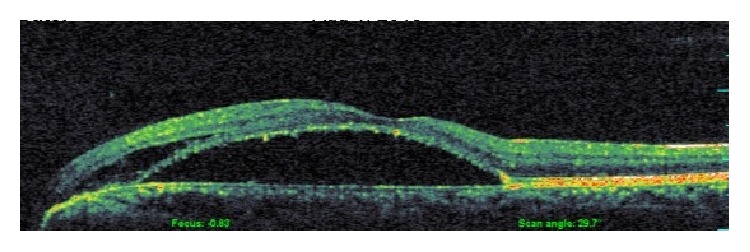


A 32-year-old Turkish male with no past history of note presented with central blurring of vision in his left eye for two months. His best-corrected visual acuity on the Snellen chart was 20/20 in the right eye and 20/200 in the left eye. The anterior segment examination was unremarkable and the intraocular pressure on Goldmann applanation tonometry was 17 mmHg on both eyes. A dilated fundus examination of the left eye revealed serous macular detachment (Figure 1). An optic disk pit was seen in the inferotemporal aspect of the optic disc continuous with the area of retinal detachment. The optic disk and fundus of right eye were normal. The optic disk and fundus of right eye were normal. The spectral domain optical coherence tomography (Opko/Oti Oct (Ophthalmic Technologies Inc., Toronto, Canada)) revealed serous macular detachment and retinoschisis. Central macular thickness was noted as 473 microns (Figure 2).

A 23-gauge pars plana vitrectomy with triamcinolone-assisted removal of posterior hyaloid interface was performed. After completing vitrectomy, internal limiting membrane peeling and fluid-air exchange were performed. Peripapillary endolaser barrage photocoagulation was performed temporally, and air-gas exchange was performed with perfluoropropane (C3F8) gas. The patient was instructed to keep prone for one week.

**Figure 3:**
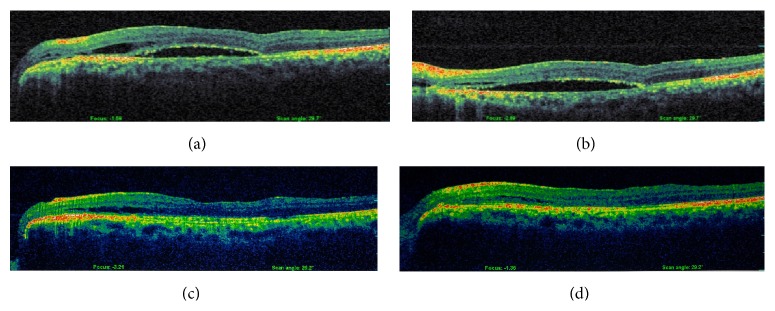


During the next six months the retina gradually flattened (Figure 3) and this was followed by a gradual improvement of BCVA to 20/30 in the left eye. At 18 months SD-OCT showed minimal subretinal fluid and BCVA was 20/40 (Figure 4).

## 3. Discussion

Optic disc pit is an uncommon congenital anomaly, usually associated with macular serous retinal detachment [2].

The source of the subretinal fluid is controversial. It is postulated that the possible sources of intraretinal/subretinal fluid might be vitreous cavity [4], cerebrospinal fluid from the subarachnoid space [5], and leaky blood vessels at the base of the optic disc pit [6]. How this fluid tracks into the retina is unknown. But most likely the fluid initially forms a schisis and subsequently enters the subretinal space and creates a less extensive detachment of the outer retina [2].

Twenty-five percent of cases with maculopathy secondary to optic pit resolve spontaneously [7]. However, the poor visual outcome of conservative management has prompted use of a more aggressive approach [8].

There is no consensus of the treatment of maculopathy secondary to optic pits. The treatment options range from barrage laser photocoagulation to vitrectomy, with or without adjunctive procedures such as internal limiting membrane (ILM) peel, gas tamponade, and laser photocoagulation. Shukla et al. performed vitrectomy with ILM peeling, barrage laser photocoagulation, and gas tamponade in their study [9]. Good visual outcomes were achieved; however, more than half of the patients developed full-thickness macular holes postoperatively. The authors attributed the high incidence of FTMH to the peeling of ILM over thinned-out retina. In our case the same surgical technique was used and during follow-up period, minimal recurrence of subretinal fluid was observed.

Vitrectomy with or without internal limiting membrane peel, with or without gas tamponade, and with or without endolaser photocoagulation has also been reported to improve vision. Although there are several treatment options, none of them has been accepted as the best treatment method. Our treatment includes vitrectomy, posterior hyaloid and internal membrane peeling, gas tamponade, and laser photocoagulation. Some authors prefer surgical treatment without laser photocoagulation; for example, Hirakata et al. reported the success of vitrectomy with induction of posterior vitreous detachment and gas tamponade, without additional laser treatment in reattaching the macula, and improvement in central vision in most patients with optic disc pit maculopathy [10]. They suggested that peripapillary vitreous traction with the passage of fluid into the retina through the pit is the cause of the schisis-like separation seen in optic disc pit maculopathy. On the other hand some authors add laser photocoagulation to their surgical method. Avci et al. also performed pars plana vitrectomy, posterior hyaloid removal, endolaser photocoagulation, and C3F8 gas tamponade and provided high rates of anatomical and visual success [11]. We chose argon laser photocoagulation because photocoagulation can block the abnormal communication between the pit and the adjacent inner retinal layers and is thus critical in reducing the inflow of fluid from the pit to the macula. In the absence of strong evidence for any particular treatment, it may be wise to follow a graded approach, starting with argon laser photocoagulation and progressing to surgery if the maculopathy fails to resolve. It is also unknown whether morphological features, such as multilayered schisis, outer retinal dehiscence, or neurosensory detachment, may necessitate earlier or more aggressive treatment for better visual outcome.

In conclusion, vitrectomy combined with posterior hyaloid and internal limiting membrane peel from the macular area, followed by air tamponade, with additional laser photocoagulation was successful for the treatment of optic disc pit maculopathy in our patient. Further studies are needed to explore the significance and impact of structural features in optic disc pit maculopathy on the choice of treatment and visual prognosis.

## Figures and Tables

**Figure 4:**